# Ocular complications in HIV positive patients on antiretroviral therapy in Ghana

**DOI:** 10.1186/s12886-016-0310-5

**Published:** 2016-08-03

**Authors:** Alexander Martin-Odoom, Evelyn Yayra Bonney, Derek Kofi Opoku

**Affiliations:** 1 Department of Medical Laboratory Sciences, School of Biomedical & Allied Health Sciences, College of Health Sciences, University of Ghana, Accra, Ghana; 2 Department of Virology, Noguchi Memorial Institute for Medical Research, College of Health Sciences, University of Ghana, Accra, Ghana

**Keywords:** Human immunodeficiency virus, Anterior segment, Visual impairment

## Abstract

**Background:**

Patients infected with human immunodeficiency virus (HIV) usually develop some form of ocular complication in the different segments of the eye due to immune deficiency. In Ghana, data regarding ocular complications among HIV/AIDS patients is scarce. This study investigated the occurrence of ocular complications in HIV infected patients undergoing antiretroviral therapy at the Agogo Presbyterian Hospital in the Ashanti Region of Ghana.

**Methods:**

Blood samples were taken from 100 confirmed HIV infected patients. The CD4 + T cell count and WHO clinical staging were determined. The patients were taken through thorough ophthalmic assessments to determine any ocular complications.

**Results:**

Forty-eight patients (48 %) had at least one HIV-related ocular complication. These complications occurred more frequently among those with CD4 counts below 200 cells/μL. Of the participants with HIV-related ocular complications, 11 (23 %) had retinal microvasculopathy, 10 (21 %) showed allergic conjunctivitis, 7 (15 %) had HIV retinopathy and 7 (15 %) had conjunctival carcinoma. All the participants in the study were on first-line antiretroviral therapy; 68 % were females and 72 % were in the Stage 3 of the WHO Clinical Staging of HIV infection.

**Conclusion:**

The prevalence of ocular complications in HIV positive persons under treatment in Ghana is high. Lower CD4 + T cell counts coupled with age were predisposing factors to HIV-related ocular complications.

## Background

HIV infection can lead to Acquired Immune Deficiency Syndrome (AIDS). The infection leads to the gradual decrease in CD4+ T lymphocytes causing subsequent opportunistic infections and neoplasia. Up to 70 % of patients infected with HIV have been found to have developed some form of ocular complications [1].

The CD4 + T lymphocytes count has been used to predict the onset of certain ocular infections in HIV positive patients. A CD4 + T lymphocytes count below 100 cells/μL is associated with retinal or conjunctival microvasculopathy, Cytomegolovirus (CMV) retinitis, Varicella – Zoster Virus retinitis, cryptococcosis, microsporidiosis, HIV encephalopathy and progressive multifocal leuco-encephalopathy [2]. The introduction of highly active antiretroviral therapy (HAART) has led to a decrease in the incidence of ocular opportunistic infections causing retinitis in HIV positive persons as a result of improved immune status [3]. Prior to the introduction of HAART, CMV retinitis affected 30–40 % of HIV-infected individuals, with visual loss primarily due to CMV involvement of the posterior retina and retinal detachment; it has also been suggested that upon careful examination, 30 % of patients with CD4 + T lymphocytes counts below 50 cells/μL would be suffering from CMV retinitis [4].

Ocular complications in HIV patients make management of such patients more difficult; if such manifestations can be picked up early, better management results could be achieved with such patients. The types of ocular manifestations seen in developing nations such as Ghana vary in comparison to those reported in developed countries [5]. A study of ocular complications in a hospital based in Ethiopia showed a prevalence rate of 60 % [6]. Anterior segment involvement usually results in tumours and external infections while posterior segment involvement usually results in HIV-retinopathy and a number of opportunistic infections of the retina and the choroid [7]. The posterior segment complications usually lead to severe visual impairment or blindness if not detected and treated early [8].

Many serious eye complications are also associated with advanced immunosuppression stages of the HIV infection. The more compromised the immune status of the HIV patient becomes, the more likely ocular complications can develop in the patient [7].

There is a dearth of information on the spectrum, nature and the extent of loss of vision that predisposes an HIV positive person to AIDS-related ocular complications in Ghana.

This study set out to detect the presence of ocular complications in HIV infected patients undergoing antiretroviral therapy (ART) in Ghana and to establish the relationship between the ocular manifestations and the CD4+ T cell counts in order to provide baseline information to aid in the management of HIV positive persons. For people living with HIV/AIDS (PLWHA) in Ghana on ART, the study also sought to assess the relationship between the age of HIV positive patients and occurrence of ocular complications.

## Methods

### Study design and participants

This study was a cross-sectional study carried out at the Agogo Presbyterian Hospital at Agogo Ashanti Akyem in the Ashanti region of Ghana; the hospital serves as a referral centre for numerous ophthalmological services throughout the country. Ethical issues were strictly adhered to by first obtaining ethical clearance from the Ethics and Protocol Review Committee of the School of Allied Health Sciences, College of Health Science, University of Ghana. Permission was also obtained from the hospital administration, the Counselling Unit, the Eye Clinic and the Laboratory unit before the study was carried out. The study enrolled 100 patients who had been diagnosed and confirmed as HIV positive and were undergoing antiretroviral therapy at the hospital. Voluntary written informed consent was obtained from each subject prior to inclusion in the study. Patients who were known to have ocular manifestations before being diagnosed as HIV infected were not included in the sampling.

A structured questionnaire was administered to obtain the demographic data of the study subjects, any familiar ocular manifestations, and any observed ocular manifestations since the therapy. Information on the antiretroviral regimen in use, the WHO clinical staging of the HIV infection, the length of time since initiating the therapy, and consistency in attending the health centre for the therapy were extracted from the patient’s folder at the hospital.

### Sample collection and analysis

For each patient 3 ml of venous blood sample was collected through venipuncture into EDTA - treated tubes and serologically confirmed as HIV positive. The CD4+ T cell count for each patient was determined at the hospital using the BD FACSCount equipment and detection kit.

The study subjects were taken through a thorough eye examination for each eye at the Optical Unit of the Eye Clinic of the hospital by an ophthalmic nursing officer, and duly recorded complications encountered for each eye. The parts of the eye examined included the eye lid, conjunctiva, cornea, anterior chamber, iris, pupil, lens, vitreous fluid fundus and the macula. The Visual Acuity Testing was done where the study subjects were asked to read various test alphabets and symbols in order to grade their vision. The Slit lamp was used to examine the anterior segment of the eye from the lid to the pupil of the eye. The Ophthalmoscope was used to examine the posterior segment of the eye to detect any abnormalities from the lens of the macula of the eye. All ocular related observations were recorded and any defects seen were duly diagnosed. The eye examination followed a standard protocol in place at the Eye Clinic of the hospital.

Data analysis was done using descriptive statistics and the Statistical Package for Social Sciences (SPSS) version 20 statistical software for windows. Pearson Chi – Square was used to calculate a *p* – value significant at <5 % at a 95 % confidence interval to find the relationship between any two given variables of the study.

## Results

The study characteristics of the enrolled participants are shown in Table 1. The visual acuity testing showed the participants had different vision grades though none was sight threatening, going by the hospital’s standard operational procedures. Out of the 100 participants recruited for the study, 57 (57 %) showed various ocular manifestations; however, HIV/AIDS-related ocular complications were seen in 48 % (48) of the study participants and these are depicted in Table 2. Other ocular findings not specifically related to HIV occurred in 9 % of the study population; these ocular findings were pinguecula, refractive errors, epiphora, presbyopia and early lens changes. There were no ocular manifestations found in 43 (43 %) of the study participants. Using a set *p*-value of 0.05 at a 95 % confidence interval, the association between the variables and the number of participants with ocular complications were determined; Pearson’s Chi – Square was used to calculate the *p*-values. These *p*-values are shown on Table 1.

**Table 1:** Study characteristics of participants and study associations

Characteristics		Number of participants, *n* (%)	Number with HIV-related ocular complications, *n* (%)	*p*-value*
Age group (years)	20 ≤ 30	16	7 (15)	0.005
	31 ≤ 45	60	27 (56)	
	>45	24	14 (29)	
Gender	Male	32	15 (31)	0.479
	Female	68	33 (69)	
HIV Type	1	90	42 (88)	0.074
	2	7	5 (10)	
	1&2	3	1 (2)	
WHO Clinical Staging	1	2	2 (4)	<0.001
	2	23	8 (17)	
	3	72	37 (77)	
	4	3	1 (2)	
Ranges of CD4+ T Counts (cells/μL)	0 ≤ 200	36	24 (50)	0.001
	201 ≤ 500	46	14 (29)	
	> 500	18	10 (21)	
Adherence to Treatment	Regular	74	21 (44)	0.001
	Irregular	3	3 (6)	
	Defaulting	23	24 (50)	
Antiretroviral Regimen	1^st^ choice	75	40 (83)	<0.001
	2^nd^ choice	25	8 (17)	

*Significant at 5 %; 1^st^ choice ARV regimen – Zidovudine + Lamivudine + Nevirapine/ Efavirenz; 2^nd^ choice ARV regimen – Tenofovir + Lamivudine + Nevirapine/ Efavirenz; ≤ Less than or equal to; ≥ More than or equal to; > More than; < Less than

**Table 2:** HIV/AIDS related ocular complications in study participants

Ocular complications	Number of participants, *n* (%)
Retinal Microvasculopathy	11 (23)
Allergic Conjunctivitis	10 (21)
Conjunctival carcinoma	7 (15)
HIV Retinopathy	7 (15)
Immature Cataract	5 (10)
HIV Bilateral optic atrophy	3 (6)
Herpes Zoster Ophthalmicus	2 (4)
Conjunctival Squamous Cell Carcinoma	2 (4)
Uveitis	1 (2)
TOTAL	48

Of the participants showing HIV-related ocular complications, 50 % (24/48) were found among participants with CD4 values less than or equal to 200 cells/μL; this was followed by 29 % (14/48) in the participants with CD4 counts between 201 and 500 cells/μL. Persons with CD4 counts more than 500 cells/μL showed the least proportion of HIV-related ocular complications, 21 % (10/48). The difference between the CD4 levels was significant at a *p* value of 0.001 (Table 1). The mean CD4 count for participants with no ocular manifestation was 593 cells/μL.

Study participants in the age range of 31–45 years showed the highest proportion of HIV-related ocular complications, 56 % (27/48); this was followed by 29 % (14/48) seen in participants above 45 years of age, then 15 % (7/48) seen in the age range of 20-30years. The difference between the age groups was significant at a *p* value of 0.005 (Table 1). The majority of the participants, 77 % (37/48), with HIV-related ocular complications were in the WHO Clinical Stage 3 of the disease; persons with HIV-related ocular complications in Clinical Stage 2 made up 17 % (8/48) with Clinical Stages 1 and 4 sharing up the rest, 4 % (2/48) and 2 % (1/48) respectively. The difference in the proportions among the WHO Clinical Stages was significant at *p* <0.001 (Table 1).

The differences in the proportions of male and female who had HIV-related ocular complications and the type of HIV involved were not significant at *p* values > 0.05 (Table 1).

Ocular manifestations occur in various parts of the eye: the anterior, the posterior and the adnexal segments. In this study in Ghana, the HIV-related ocular complications found in the adnexal and anterior segments included conjunctival carcinoma, allergic conjunctivitis, uveitis, immature cataract, Herpes Zoster Ophthalmicus and conjunctival squamous cell carcinoma. Epiphora, pinguecula, refractive error, presbyopia and early lens changes were also seen in these segments, though these are not HIV-related findings. HIV-related ocular complications seen in the posterior segments included bilateral optic atrophy, retinal microvasculopathy and HIV retinopathy, all of which affect the retina of the eye. Table 2 depicts the HIV- related ocular complications encountered in this study.

Through the use of the structured questionnaire the study established that 74 % of the participants regularly adhered to treatment whilst 23 % of participants defaulted between two (2) weeks and 52 weeks (1 year). Only 3 % of participants were irregular in adhering to treatment. However, 44 % (21/48) of participants with ocular complications regularly adhered to treatment whilst 50 % (24/48) of those with HIV-related ocular findings defaulted in their treatment for periods between 2 weeks and 52 weeks (1 year). Those participants with HIV-related ocular complications who were irregular in adhering to treatment made up 6 % (3/48) as shown on Table 1. From data obtained from the participants using the structured questionnaire, a *p* value of 0.583 was calculated for the association between the length of time a participant had been on ART and the occurrence of HIV-related ocular complications.

## Discussion

With the scaling up of antiretroviral drugs availability and use in Ghana, people living with HIV/AIDS (PLWHA) have a chance of having a better quality of life as compared to the pre-antiretroviral therapy period. Ocular complications in PLWHA would subsequently become an issue worth paying attention to for the general good health of HIV positive persons under treatment in Ghana.

The prevalence rate of HIV-related ocular complications determined in this study was 48 %. This prevalence rate is quite similar to that seen in a study done by Assefa et al. in Ethiopia in the year 2006 that showed a prevalence rate of 60 % [6]. The prevalence registered in some other countries are comparable to that of this study though some had lower prevalence values than the present study [9–13] as shown in Table 3. In this study in Ghana, the commonest ocular complication seen was retinal microvasculopathy (23 %) as depicted in Table 2; this is similar to work done in Ethiopia [6] where retinal microvasculopathy was found in 24 % of the HIV positive persons of the study. In other African countries retinal microvasculopahty was also found to be the most common HIV-related ocular complication, ranging from 10 % to 42 % of the study persons [14, 15].

**Table 3:** Comparison with Studies done in different countries

Study	Jabs [9], 1995, USA	Awan et al. [10], 1996, Kenya	Biswas J et al. [11], 2000, India	Sriprakash K. S. et al. [12], 2001–2003 India	Purushottam J et al. [13], 2006, Nepal	Present study, 2013, Ghana
Sample size	781	102	100	175	103	100
Male %	88	62	77	54.2	68.9	32
Female %	12	38	23	45.8	31.1	68
HIV-related Ocular complications %	50	66	40	40.57	38.8	48

In this study, the ages of the 100 participants enrolled in the study ranged from 20 years to above 60 years. Though HIV-related ocular complications were diagnosed in all the age groups, those in the 31–45 years range were determined as the most susceptible to the development of ocular complications (56 %) followed by persons in the study who were older than 45 years (29 %) as shown in Table 1. The difference in the proportions ascribed to the age groups was significant. Thus PLWHA in the older age brackets in Ghana had a higher possibility of developing ocular complications. This trend would be seen regularly as antiretroviral use contributes to the increasing life span of the HIV positive persons and they live longer.

The WHO Clinical Staging of HIV infection is based on the immune status of the patient and the signs and symptoms shown upon initial diagnosis in order to monitor the disease process. In this study the majority of the participants who exhibited HIV-related ocular complications (77 %) were in the WHO Clinical Stage 3 of the disease (Table 1) which is a later stage of HIV infection. The situation could be the underlying factor for the quite high prevalence of ocular complications found in this study. There is a significant association between the clinical stage of the disease and the ocular complications development as shown by a calculated *p* – value less than 0.001. Thus the HIV-positive persons become more susceptible to the development of some form of ocular complications as the infection progresses. This is similar to the results obtained by Ahmed et al. in 2005, which showed that eye problems are associated with the advanced immunosuppression stage of the HIV infection [7].

There is a significant association (*p*-value of 0.001) between the development of ocular complications and the CD4+ T cell count of the patient. Majority of participants 50 % (24/48) in this study diagnosed with ocular complications had CD4+ T cell counts less than 200 cells/μL; this indicates that a CD4+ T –cell count of less than 200 cells/μL predisposes HIV patients much more to the development of ocular complications. The ocular complications associated with CD4+ T cell counts less than 200 cells/μL were more, compared to those with higher counts. The most prominent of those complications were retinal microvasculopathy, conjunctival growth, HIV retinopathy and allergic conjunctivitis. The lower the CD4 + T cell count, the more susceptible the HIV patient is to the development of any ocular complications. This is in line with findings that CD4+ T cell count less than 100 cells/μL is associated with retinal or conjunctival complications shown by Robert et al. [2]. A higher CD4+ T cell count gives a better chance of suppressing the involvement of ocular complications; this is supported in this study by lesser proportions of ocular involvement recorded in participants with CD4 + T cell count above 500 cells/μL and supported with the findings in this study that indicated that those participants with no HIV-related ocular complications had higher CD4 + T cell counts.

Adherence to treatment significantly reduces the development of ocular complications in the HIV patients who are regular for treatment as seen in this study (Table 1). Participants who defaulted in adhering to their treatment made up 50 % of those with ocular complications as per Table 1. A *p*-value of 0.001 indicated the significant association between the categories of patients adherent to treatment and the occurrence of ocular complications. The more regular a patient is in adhering to the antiretroviral therapy (ART), the less likely they are to develop ocular complications. This inference is in consonance with work done by Kumarasamy et al., in 2005 which showed the use of ART had decreased the occurrence of ocular manifestations in HIV positive patients [3]. The use of antiretroviral therapy has led to the decline of opportunistic infections such as Cytomegalovirus retinitis in HIV-positive persons since the progress of the infection to the immune-deficient stage is moderated by the drugs [16, 17]. In this study the absence of opportunistic infections could be due to the scaled-up status of antiretroviral drugs availability, increased use of ARVs and the improved immune status of the HIV-1 positive patients. This situation is in consonance with work done on the epidemiology of CMV retinitis in Africa among HIV/AIDS patients in which it was established that the low prevalence of CMV retinitis in Africa compared to Europe and the United States indicated that most African HIV/AIDS patients on treatment have an improved life span and die from other diseases before they enter the stage of severe immune depression associated with CMV disease [18]. The majority of the participants in this Ghanaian study had CD4 levels above 200 cells/μL (Table 1). This could also explain the absence of blinding CMV retinitis among the Ghanaian HIV/AIDS patients.

This study determined that gender difference does not impact on the occurrence of ocular complications in PLWHA- both female and male HIV patients were equally susceptible to the developement of ocular complications. Thus there was no statistically significant difference between the occurrence of ocular complications and the gender of the participants; *p* – value was 0.479 (Table 1).

In this study the occurrence of ocular complications in HIV positive persons is shown not to depend on the HIV type. It has been shown in another study that HIV Type 1 is more virulent than HIV Type 2 [19], however, in this study there was no significant difference between the type of HIV infection and the development of ocular complications in the study subjects (*p* – value was 0.074).

This study determined that there is no significant association between the development of HIV-related ocular manifestation and the length of time a PLWHA had been on antiretroviral therapy so far as predisposing factors like CD4 + T cell count and age are favourable. With regards to the antiretroviral regimen in use all the participants were on first line drugs; however, those on the first line first choice option drugs (Zidovudine + Lamivudine + Nevirapine/Efavirenz) showed a higher proportion of participants (83 %) with ocular complications as compared to those on the first line second choice option drugs (Tenofovir + Lamivudine + Nevirapine/Efavirenz) who had a smaller proportion with HIV-related ocular involvement (17 %). This difference is significant with a *p*-value less than 0.001.

This study is pointing to the positive effect the first line second choice option drugs could have on minimizing ocular involvement among PLWHA on ART in Ghana. A larger study would be necessary to adequately examine this possibility and its impact on the management of HIV positive persons with ocular manifestations.

## Conclusion

The prevalence of ocular complications in HIV positive persons under treatment in Ghana is high. Lower CD4 + T cell counts coupled with older age makes the HIV positive person more susceptible to ocular complications. The study findings make it imperative for mandatory comprehensive ophthalmologic assessment of the HIV/AIDS patient in Ghana to be instituted in view of the scaled-up antiretroviral therapy programme. This would ensure early detection and treatment of ocular involvement in the PLWHA as the disease progresses.

### Limitation of the study

Lack of baseline information on the ophthalmic assessment upon initial diagnosis of HIV infection, limited the study in relating the appearance of ocular involvement with the early stages of the HIV infection.

## Abbreviations

AIDS, acquired immune deficiency syndrome; ART, antiretroviral therapy; CD4, cluster of differentiation type 4; HAART, highly active antiretroviral therapy; HIV, human immunodeficiency virus; PLWHA, people living with hiv/aids; WHO, World Health Organization
